# From Joint Thinking to Joint Action: A Call to Action on Improving Water, Sanitation, and Hygiene for Maternal and Newborn Health

**DOI:** 10.1371/journal.pmed.1001771

**Published:** 2014-12-12

**Authors:** Yael Velleman, Elizabeth Mason, Wendy Graham, Lenka Benova, Mickey Chopra, Oona M. R. Campbell, Bruce Gordon, Sanjay Wijesekera, Sennen Hounton, Joanna Esteves Mills, Val Curtis, Kaosar Afsana, Sophie Boisson, Moke Magoma, Sandy Cairncross, Oliver Cumming

**Affiliations:** 1 WaterAid, London, United Kingdom; 2 University College London, London, United Kingdom; 3 London School of Hygiene & Tropical Medicine, London, United Kingdom; 4 University of Aberdeen, Aberdeen, United Kingdom; 5 The SoapBox Collaborative, Aberdeen, United Kingdom; 6 UNICEF, New York, United States of America; 7 World Health Organization, Geneva, Switzerland; 8 United Nations Population Fund, New York, United States of America; 9 BRAC and BRAC University, James P Grant School of Public Health, Dhaka, Bangladesh; 10 Evidence for Action (E4A), Bugando Consultant, Teaching Hospital, Dar es Salaam, Tanzania

## Abstract

Yael Velleman and colleagues argue for stronger integration between the water, sanitation, and hygiene (WASH) and maternal and newborn health sectors.

*Please see later in the article for the Editors' Summary*

**Summary Points**

- There is sufficient evidence that water, sanitation, and hygiene (WASH) may impact maternal and newborn health (MNH) to warrant greater attention from all stakeholders involved in improving MNH and achieving universal WASH access.
- Enabling stronger integration between the WASH and health sectors has the potential to accelerate progress on MNH; this should be accompanied by improving monitoring of WASH in health care facilities providing MNH services as part of routine national-level monitoring, and at the global level through international instruments.
- Global and national efforts to reduce maternal and newborn mortality and morbidity should adequately reflect WASH as a pre-requisite for ensuring the quality, effectiveness, and use of health care services.
- The Post-2015 development framework is an opportunity for a stronger, more inter-sectoral response to the MNH challenge, and the goals and targets aimed at maximizing healthy lives and increasing access to quality health care should adequately embed WASH targets and success indicators.
- Further implementation research is needed to identify effective interventions to improve WASH at home and in health care facilities, and to impact on MNH in different health system contexts.

## Water, Sanitation, and Hygiene, and Maternal and Newborn Health: An Opportunity for Progress

The “deep dark and continuous stream of mortality” lamented by William Farr in 1876 when describing maternal mortality statistics in England [1] continues in many parts of the world today, and for some families, childbirth is as much a risk of death as a moment of life. Progress has been slow compared with other areas of public health, and geographically and socio-economically unequal; maternal and newborn health (MNH) remains a major global challenge [2],[3].

Newborn mortality has decreased more slowly than overall under-five mortality, and accounts for a median share of 44% of under-five mortality in high-burden countries [4]. Between 1990 and 2012, newborn mortality declined by only 37% from 33 to 21 deaths per 1,000 live births, compared with a more impressive 50% reduction in under-five mortality over the same period [4]. Progress on reducing maternal mortality has been even slower and more uneven across countries, with a median annual rate of reduction in high-burden countries between 2000 and 2013 of 3.1% [5]. Whilst the maternal mortality ratio (maternal deaths per 100,000 live births) has fallen globally from 380 to 210 since 1990 [6], these figures mask wide disparities. In 2013 the average maternal mortality ratio in developed countries was 16 per 100,000 live births compared with 230 in low- and middle-income countries (LMICs) [6]. This chasm separating the prospects of women giving birth in one part of the world as compared with another is what Halfdan Mahler, then Director General of the World Health Organization (WHO), described in 1987 as “the largest discrepancy of all public health statistics” [7].

Many calls have been made for wider and better-coordinated efforts to leverage increased resources and more effective action on MNH [8], particularly in low-income, high-burden settings [9]. Linking investments in water, sanitation, and hygiene (WASH) presents an overlooked but potentially important opportunity for progress. WASH—defined as improved water quantity and quality, sanitation, and hygiene—can prevent or limit the transmission of disease through multiple routes [10],[11]. As a sector, WASH spans a broad range of interventions, from campaigns to promote sanitation and hygiene behaviours, to water and sanitation infrastructure, to regulation of service quality and cost of drinking water or sanitation services [12].

A lack of coherence between sectors and programmes has been implicated in the poor progress on some Millennium Development Goals (MDGs) and targets (see Box 1), including the MDGs for maternal and child health [13]. Coordination between the WASH sector and the health sector is challenging; opportunities for better integration have been identified [14], although the focus is often on child health rather than maternal or newborn health [15]. Recently, growing concern about health care-associated or nosocomial infections has increased attention to hygiene in health care facilities under the “Clean Care is Safer Care” banner of the WHO Patient Safety initiative [16],[17]. The WHO guidelines on hand hygiene in health care facilities also recognise the importance of water and, to a lesser extent, sanitation as determinants of safe hand hygiene [18].

**Box 1. Review of Policy Documents in Bangladesh: 2000 to Date**

Three of the authors (KA, LB, and OMRC) conducted a case study of policy and planning within the maternal and child health sectors, and the WASH sector in Bangladesh, to elucidate the current state of synergy and linkage across sectors. Policy documents from the Bangladesh Ministries of Health and Family Welfare, Water and Sanitation, Food and Disaster Management, Education, Finance, and Foreign Affairs, and the Department of Public Health Engineering, were screened to identify whether any linkages between maternal, neonatal, and reproductive health and WASH were mentioned. In general, policy and programmes in WASH and maternal health were not connected for enhancing wider opportunity and synergistic impact. WASH documents made passing reference to improving maternal and child health, but surprisingly did not advocate for adequate sanitation or water in health care facilities (although they did mention bus stations, markets, schools, and mosques). Recently, the 2011–2016 Bangladesh Health Population and Nutrition Sector Development Programme mentioned that “facilities will be user and women friendly with adequate arrangements for female toilets, hand washing, water and sanitation.” The 2007 National Strategy for Infant & Young Child Feeding in Bangladesh mentioned the need for drinking water for pregnant and lactating women, while the 2009 National Neonatal Health Strategy and Guidelines For Bangladesh mentions the need for both soap and water for handwashing, and water for mother and companion. The review suggested that explicit links (e.g., need for WASH in health care facilities) are relatively recent and limited in scope.

Significant progress has been made on extending access to water under the MDGs, with less progress on sanitation [19]. The MDG target on water and sanitation did not include access to WASH in health care facilities and other settings where births occur [20]; this has impeded the potential contribution of WASH to MNH efforts.

This collaborative paper by academics and representatives from international WASH and MNH agencies urges action and offers recommendations to accelerate WASH service provision at home and in health care facilities to improve MNH.

### The Potential Contribution of WASH Efforts to Maternal and Newborn Health

Historically, the connection between WASH and MNH is well established [21]–[23]. In 1795, Alexander Gordon (1752–1799) asserted that deaths from puerperal fever could be prevented with greater cleanliness and that “nurses and physicians ought carefully to wash themselves” after contact with an infected patient [21]. Ignaz Semmelweis (1819–1865) later achieved a dramatic reduction in maternal deaths by requiring doctors to wash their hands in chlorine solution before examining women in labour [24].

By modern standards, there is a dearth of rigorous research to quantify the effects of WASH interventions on MNH outcomes. A recent systematic review of the association between water and sanitation environments and maternal mortality found only 14 relevant studies, none of which were intervention studies [25]. Although all studies had limitations, a pooled analysis of those linked at an individual level (case-control design) found that poor water and sanitation access was associated with higher levels of maternal mortality. A study by Gon and colleagues showed that unimproved household water access was an important risk factor for pregnancy-related mortality in Afghanistan [26].

Although no systematic review could be identified on the effect of WASH on neonatal mortality, a recent systematic review and Delphi estimation found that “clean birth practices” in both homes and facilities were associated with reduced all-cause, sepsis and tetanus neonatal deaths [27]. The review did not consider water and sanitation access in birth environments, but eight observational studies concerning handwashing with soap by birth attendants were included and all were consistently protective for neonatal sepsis and cord infection [27]. One cohort study in Nepal found that birth attendant and maternal handwashing were protective against neonatal mortality, with a 41% (95% CI 6%–63%) lower mortality rate among neonates exposed to both practices [28].

### The Current Challenge

**Figure 1:**
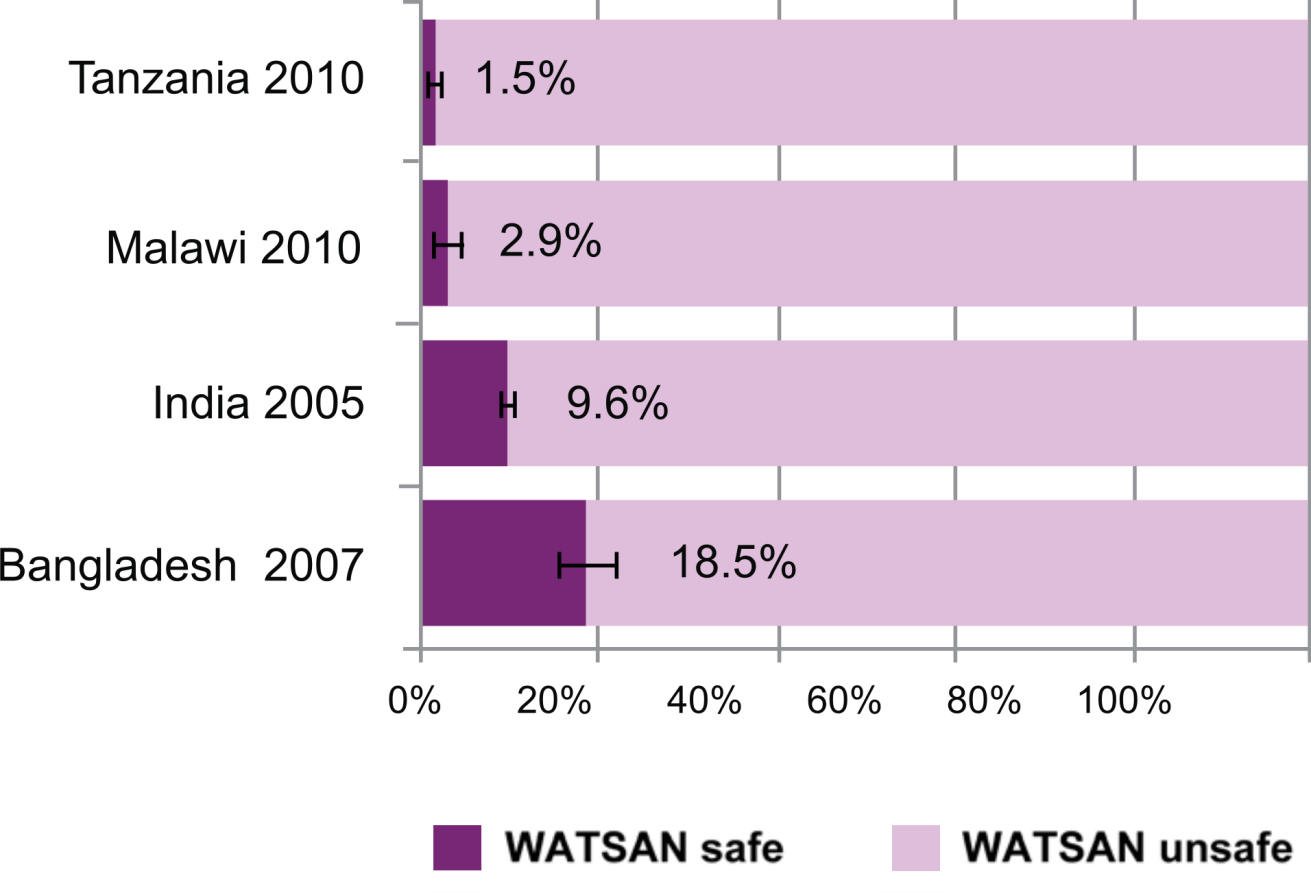
Proportions of births occurring in current household environments in the five years preceding the survey, by type of WATSAN environment. Error bars represent 95% confidence intervals of estimates. Definitions: Birth environments were defined as “WATSAN safe” or “WATSAN unsafe,” rather than “WASH safe”/“WASH unsafe.” WATSAN-safe was defined as the availability of and access to improved water sources and improved sanitation facilities, but not including hygiene practices, water quality, or consistency of availability. Source: Demographic and health surveys (DHS) data for the four countries shown (year of survey in parentheses); analysis as described by Benova and colleagues [30].

While the importance of hygiene is increasingly being recognized, far less consideration has been given to the role of the complete WASH package in relation to MNH outcomes in both home and facility birth settings. A recent WHO rapid assessment of WASH coverage in health care facilities in 54 low-income countries found that 38% of these facilities lacked an available improved water source [29]. In some low-income settings, many more women give birth in domestic environments than in health care facilities, and these are often without any basic water and/or sanitation. Figure 1 shows estimates for the proportion of births that occur in homes without improved water and/or sanitation for four countries (Bangladesh, India, Malawi, and Tanzania) [30]. These countries were selected as they are the focus for an on-going research programme (the SHARE research consortium); they also provide case studies from the two regions that have the lowest levels of WASH coverage and highest maternal and neonatal disease burden (sub-Saharan Africa and South Asia). In all four countries, only a minority of home births occur in environments where adequate water and sanitation are available. This is of major importance in low-income settings where the burden of health care-associated infections is potentially much higher [31], as is maternal and newborn mortality.

Efforts to improve birth conditions in low-income countries have tended to focus on specific measures for maternity care, health system strengthening, and increasing women's demand for giving birth in health care facilities [32],[33]. Little attention has been paid to the conditions in which births take place. Increasing the use of health care facilities for childbirth without considering the availability and quality of WASH in these facilities may limit potential health gains. Current WHO recommendations on postnatal care for mothers and newborns [34] include only one reference to WASH, which relates to the need for counselling women on hygiene. The guidelines for the Standards for Maternal and Neonatal Care [35] include no recommendations on WASH service provision. The six essential “cleans” proposed by WHO during childbirth imply the importance of WASH but are not explicit [35]. Inevitably, health care facilities are often managed around the provision and improvement of diagnostic and treatment services, and WASH may be such an obvious requirement that it is insufficiently emphasized in national health standards and monitoring instruments. This neglect is compounded by the lack of clarity on who—within the overall structure of the health system and in individual health care facilities—should be responsible for ensuring adequate WASH provision.

Beyond the increased risk of infection where WASH is absent, a lack of drinking water or availability of safe sanitation facilities in hospitals and clinics may discourage women from giving birth in these facilities and/or contribute to delays in seeking care. The absence of basic WASH infrastructure in health care facilities may also contribute to staff absenteeism as has been found in studies from India, Indonesia, Uganda [36], and Bangladesh [37]. Further, as noted in the 2006 World Health Report, “no matter how motivated and skilled health workers are, they cannot do their jobs properly in facilities that lack clean water…” [38].

The MDGs—especially MDG5 on improving maternal health—have certainly created momentum, by emphasising the need for explicit programmes to improve maternal health. However, their siloed nature has left little room for much needed cross-sectoral collaboration and comprehensive, integrated programming across the continuum of care. The absence of targets on water and sanitation services in strategies for achieving MDGs 4 and 5 has constrained progress on reducing maternal and newborn mortality. While the drive to increase women's demand for delivering in health care facilities is needed, the benefits for MNH are compromised if these cannot provide even minimum sanitary and hygiene standards.

## A Vision for Improved Maternal and Newborn Health through Improved WASH: What Would Change Look Like?

The multiple and interrelated causes of maternal and newborn deaths each require a number of interventions [39], and no single intervention will reduce mortality significantly. Nonetheless, as WASH underlies many of the determinants as well as responses to MNH, it is an important part of a well-functioning health system that harnesses synergies between different interventions and responds effectively to MNH challenges. Box 2 details some of the lessons that can be learned from the education sector in terms of improving cross-sectoral action.

**Box 2. Cross-sectoral Action: Lessons from WASH and Education**

An international framework that reflects the complex determinants of MNH must be applied at country level to achieve results. Policies to increase women's demand for giving birth in health care facilities have parallels with free primary education policies. Successful free primary education policies operate alongside a commitment to working together with the WASH sector, so that more school WASH facilities are built to keep pace with increased school attendance. The experience in Malawi, where the Ministry of Education added data collection on WASH in schools to existing national education surveys, presents a good example of cross-sectoral collaboration [46]. This cross-sectoral collaboration goes beyond just building facilities, in order to ensure the necessary behaviour change. In the Sri Lankan district of Ampara the introduction of student brigades had a significant impact on hygiene behaviour change, contributing significantly to the appropriate use of the WASH facilities provided [46]. Similar collaborations between the health and WASH sectors at various levels in-country are vital to ensuring an improved WASH environment that could contribute to improved MNH outcomes.

The current debate on formulating a post-2015 development framework to replace the MDGs provides an opportunity to redress the currently fragmented approach to improving MNH. For a new framework to be successful it must embed time-bound targets on the underlying determinants of poor MNH outcomes, as well as the quality of services, in any new goal on improving these outcomes. This must include targets on WASH at household and facility level, and provide for cross-sectoral coordination, and joint planning, investment, and monitoring to achieve goals and targets. The recently launched WHO Every Newborn Action Plan and its accompanying WHO Quality Initiative provides a useful example of the practical application of such an approach [40].

### Building a Context for Change: Target-setting and Monitoring

A change in the way systems operate requires a change to the targets against which their performance is measured. An integrated approach to MNH will therefore require targets and indicators that mainstream WASH considerations.

#### Targets and indicators

What little time remains until the “expiry” of the MDGs should be used to maximise the potential for reaching MDG targets on MNH. Several agencies have committed to renew efforts on maternal, newborn, and child health before 2015; the World Bank, UNICEF, and Norway have announced a US$1.1 billion contribution towards meeting MDGs 4 and 5. Targeting these new resources towards ensuring that all facilities in which deliveries take place have adequate WASH provision will allow immediate action on the gaps identified in this paper. The WHO's “Essential Environmental Health Standards in Health Care” issued in 2008 (Box 3) set out what adequate provision means, and all efforts should strive towards their implementation.

**Box 3. The World Health Organization's Essential Environmental Health Standards in Health Care**

This document issued by the WHO in 2008 [47] sets out the essential environmental health standards required for varying levels of health care settings in medium- and low-resource countries. It enables health managers and planners, architects, urban planners, water and sanitation staff, clinical and nursing staff, carers and other health care providers, and health promoters to assess prevailing situations and plan the improvements that are required; develop and reach essential safety standards; and support the development and application of national policies.

The Standards contain a set of 11 **guidelines**, with a set of indicators and guidance notes and checklist for assessing the implementation of each guideline.

1. **Water quality:** Water for drinking, cooking, personal hygiene, medical activities, cleaning, and laundry is safe for the purpose intended.
2. **Water quantity:** Sufficient water is available at all times for drinking, food preparation, personal hygiene, medical activities, cleaning, and laundry.
3. **Water facilities and access to water:** Sufficient water-collection points and water-use facilities are available in the health care setting to allow convenient access to, and use of, water for medical activities, drinking, personal hygiene, food preparation, laundry, and cleaning.
4. **Excreta disposal:** Adequate, accessible, and appropriate toilets are provided for patients, staff, and carers.
5. **Wastewater disposal:** Wastewater is disposed of rapidly and safely.
6. **Health care waste disposal:** Health care waste is segregated, collected, transported, treated, and disposed of safely.
7. **Cleaning and laundry:** Laundry and surfaces in the health care environment are kept clean.
8. **Food storage and preparation:** Food for patients, staff, and carers is stored and prepared in a way that minimizes the risk of disease transmission.
9. **Building design, construction, and management:** Buildings are designed, constructed, and managed to provide a healthy and comfortable environment for patients, staff, and carers.
10. **Control of vector-borne disease:** Patients, staff, and carers are protected from disease vectors.
11. **Information and hygiene promotion:** Correct use of water, sanitation, and waste facilities is encouraged by hygiene promotion and by management of staff, patients, and carers.

Adequate targets and indicators should be formulated within the post-2015 framework. As a starting point, building on the emerging consensus among stakeholders involved in discussions on a post-2015 agenda for WASH, a target to achieve universal (total) access to WASH by 2030 is ambitious yet realistic. This target includes complete access in institutions and public spaces, such as health care facilities (see Box 4). Although such a target will be important for galvanising political will and investment, it will not on its own challenge the often siloed approach prevalent under the MDGs in which WASH is seen as separate to health, and therefore not an area of shared responsibility across sectors. Therefore, in addition to a target on universal coverage, the post-2015 framework should embed specific targets and/or indicators on access to WASH under goals and targets on improving health outcomes such as MNH. For example, a goal on reducing maternal mortality can include indicators on household water and sanitation access (determinants), as well as on WASH provision in delivery facilities (services).

**Box 4. Proposed Water, Sanitation, and Hygiene Target for the Post-2015 Development Framework**

A comprehensive consultation across the international WASH sector involving more than 100 experts from more than 60 organizations worldwide has resulted in a proposed shared vision for the Post-2015 agenda [42].

The vision is that of **universal access to safe drinking water, sanitation, and hygiene.** The proposed target to deliver this vision is, by 2030:

1. to eliminate open defecation;
2. to achieve universal access to basic drinking water, sanitation, and hygiene for households, schools, and health care facilities;
3. to halve the proportion of the population without access at home to safely managed drinking water and sanitation services; and
4. to progressively eliminate inequalities in access.

Further, WASH indicators can be incorporated into certain elements of the Universal Health Coverage (UHC) framework, which has featured prominently within discussions on health in the post-2015 framework (see Box 5). UHC includes universal population coverage, financial risk protection, and a package of services comprising prevention (including environmental health and behaviour change promotion) and treatment (curative and rehabilitative/palliative) elements. A target on UHC can include WASH elements both under prevention aspects (e.g., monitoring WASH access indicators at the community level and linking to MNH service planning, and incorporation of hygiene and sanitation promotion within health programmes), and treatment aspects (e.g., adoption and implementation of WASH standards for health care facilities in terms of both infrastructure and practices).

**Box 5. WASH and Universal Health Coverage: Embedding WASH in Health Care Services**

The emerging consensus on the need for a UHC approach presents an important opportunity to bridge the gaps between the WASH sector and the health system [48]. This approach aims to “ensure that all people have access to health information and services of sufficient quality without risk of financial hardship.” UHC, including access to universal sexual and reproductive health, is seen by many as an important aspect of the post-2015 framework, because it provides a mechanism to deliver improved health outcomes and sustainable development. It also offers an important opening for ensuring that WASH is a key component of health care. The UHC framework has the potential to become a uniting vision that brings together multiple actors and sectors in an effort to improve health outcomes; but realising this vision requires paying close attention to the *quality* of coverage as much as to the *breadth* of coverage. Specifically, WASH can and should be embedded as an important element under each of the pillars of UHC:

**Prevention:**

- **Promotive services:** promotion of safe sanitation, hygiene, and water quality and storage practices at the community and facility levels
- **Preventive services:** embedding WASH as an integral element of disease-control and nutrition programmes

**Treatment:**

- **Curative services:** improving WASH in health care facilities settings to reduce infection transmission and improve overall quality of care and service utilisation
- **Rehabilitative/palliative services:** embed WASH aspects in facility- and home-based care for chronic conditions

#### Monitoring progress indicators on WASH access and quality of care

A successful international framework that adequately addresses MNH must be accompanied by a robust system for gathering information and monitoring progress. An essential step toward the inclusion of WASH indicators in relevant monitoring frameworks will involve WASH facility monitoring within the health care delivery environment. WASH indicators currently captured in national emergency obstetric and newborn care needs assessments include the presence of a water filter or other means to make potable water available to patients and staff; functioning running water supply; and availability of chlorhexidine (proxy for disinfectants and antiseptics) [41]. However, this information is often inadequately and inconsistently captured in existing monitoring frameworks.

There are several ways to address this shortcoming. Firstly, existing data and methods for data gathering can be used more effectively. For example, using Tanzania as a case study, Benova and colleagues suggest a method through which available survey data could be used to estimate the water and sanitation environment of home and facility birth settings [30]. The authors used existing household and facility survey data to characterise home and facility birth environments as water and sanitation (WATSAN)-safe or -unsafe, and to describe the proportion of all births (home and facility) occurring in a WATSAN-safe environment. On average, 44% of health care facilities that conduct deliveries were WATSAN-safe but only 24% of delivery rooms within these facilities were WATSAN-safe. Furthermore, even if all home births took place in facilities, only 59% of all births would occur in a WATSAN-safe environment. The approach used for the analysis of the Tanzania data showed that it is possible in this way to estimate the WASH conditions under which births take place at home and in health care facilities, and that existing data collection mechanisms can be used without the need for significant redesign. Small adaptations in the ways in which data are interpreted can also help identify geographic disparities in access to WASH to assist in planning and budgeting processes. Such aspects can be incorporated into existing global monitoring platforms such as the WHO/UNICEF Joint Monitoring Programme (JMP) on Drinking Water and Sanitation [42] and Countdown 2015 [5].

Secondly, existing instruments can and should be improved to deliver on a more ambitious MNH agenda. The Service Availability Readiness Assessment (SARA) tool could help identify where the need is the greatest, but it must be strengthened in order to do so. At present, SARA evaluates a facility's water provision only; future iterations should include all relevant WASH aspects in and adjacent to maternity facilities, for staff and for patients. Recent efforts by WHO and partners to develop a global strategy on WASH in health care facilities [43] are encouraging in this respect and should be backed up with international and national support. The strategy will aim to encourage country implementation of existing standards [44] and good practice; promote expanded monitoring of WASH in health care facilities, including through strengthening existing instruments like SARAs; and based on this evidence, carry out advocacy to reverse the neglect of this crucial service quality aspect. This strategy links with efforts to ensure minimum basic infrastructure and hygiene services, including access to energy and health care waste management. The strategy will be accompanied by a specific action plan committed to by participating stakeholders. This promising new initiative should complement and bring together relevant WHO programmes and strategies such as Family Health (including MNH), health systems strengthening, and Patient Safety (covering health care acquired infections and Infection Prevention and Control).

## Delivering Good Maternal and Newborn Health through Linkages to WASH: Taking Action

Given the proven as well as potential links between WASH and MNH, we argue that an increased focus on WASH can pay dividends in terms of improved service quality; this in turn can contribute to improvements in service utilisation, and ultimately better health outcomes. It is clear that there is no time to lose given the relatively slow progress on MNH, and that current approaches insufficiently address the magnitude of the challenge. As shown in the Sierra Leone case study in Box 6, much can be achieved even in a resource-constrained and challenging environment.

**Box 6. Sierra Leone Case: Re-orientating Maternal and Newborn Programming**

In the post-conflict period, Sierra Leone was faced with a severe scarcity of qualified health care providers and functioning health care facilities to save the lives of women and children. An Emergency Obstetric and Newborn Care (EmONC) needs assessment was carried out in 2008 and revealed alarmingly low signal function indicators [49]. The programming of traditional effective interventions such as EmONC, midwifery, and family planning was confronted with the lack of electricity, water, and basic infection control supplies in operating theatres, delivery and post-delivery rooms, and even intensive care units. Following the needs assessment, development partners working in Sierra Leone re-oriented their MNH programmes to address these bottlenecks.

Bo District Hospital, together with other hospitals (Port Loko, Makeni, Moyamba, Bo, and Kenema) received support from development partners (UNFPA, UNICEF, DFID, and others) soon after the war. The hospital lacked adequate water and lighting. Post-caesarean section wound sepsis stood at 60%, which meant that hospital stay was prolonged in some cases up to 1 month. The development partners decided to drill boreholes and erect water storage facilities at the hospital and supply a generator for the operating theatre. The theatre was rehabilitated together with the maternity and neonatal unit. Staff were trained in basic WASH principles and wound care. The results were a dramatic reduction in the post-caesarean wound sepsis from 60% to less than 10% within a period of 3 months. The consumption of antibiotics plummeted. The admission delivery rate in the Unit doubled within 6 months as patients quickly learnt that the services at the maternity unit had improved. The hospital became self-sustaining simply by charging a booking antenatal fee of SLL 5,000 (equivalent to US$1.20).

These changes had a positive impact on staff motivation. With the documented results from Bo District Hospital and advocacy efforts directed at health development partners, this intervention was replicated in eight district hospitals, including the Teaching Hospital in Freetown. Realizing the benefits of the integrated approach, Sierra Leone formed a Facility improvement Team (FIT), which formulated a set of indicators to determine the suitability of facilities to conduct safe deliveries.

There are important steps that can be taken immediately by the international and national community to address the issues raised in this paper:

1. **Support and implement the forthcoming WHO strategy on WASH in health care facilities:** We welcome this initiative and urge donors, national governments, and other agencies to adopt the proposed actions, and implement the existing standards as part of overall national action to reduce maternal and newborn mortality. Implementing the strategy will entail firstly high-level political recognition that WASH is a critical component of MNH strategies. Secondly, it will require reorienting management and budgeting priorities and standards to include the necessary infrastructure and supplies, training, and monitoring. Thirdly, simple, low-cost practices should be applied at the facility level to maintain basic hygiene and sterile conditions, particularly in delivery rooms and operating theatres.
2. **Support the implementation of the WHO Every Newborn Action Plan (ENAP) in its entirety, with a specific emphasis on WASH:** We welcome this plan and its comprehensive attention to all aspects contributing to newborn health within and outside of health care facilities. ENAP includes attention to household access to water and sanitation, WASH within the domains of quality-of-care for maternal and newborn care and infection prevention and control, and the importance of cross-sectoral action to improve newborn health. The inclusion of WASH interventions in the Every Mother Every Newborn Quality Initiative [45] will be critical for the Initiative to be effective. To ensure that the ENAP and related initiatives result in improved MNH outcomes, they must be translated into national roadmaps that adequately reflect the role of WASH in terms of financial and human resourcing, monitoring systems, and training of health care staff; and that link MNH efforts to existing national plans and programmes to improve access to WASH and improve public health.
3. **Embed WASH in national and global implementation and monitoring frameworks for Universal Health Coverage:** The drive to achieve UHC is a unique opportunity to redress the neglect of public health in recent decades, as it positions prevention and treatment side by side as core components of a well-functioning health system. WASH is crucial for the success of the UHC model as it contributes to both preventive and treatment aspects and is a core component of quality of care. Any global and national monitoring frameworks on UHC should include performance indicators on access to WASH at household and health care facility levels and across all health services. Data on these performance indicators should be routinely collected, shared, and used to plan and prioritise actions and resources.
4. **Embed WASH in the post-2015 development framework:** In this paper we proposed the various ways in which WASH should be built in to the new development framework. This integration is a crucial opportunity to address the shortcomings of existing goals and targets and encourage cross-sector action to improve health outcomes through addressing WASH in both domestic and health care facility settings. We call on all stakeholders engaged in discussion on the post-2015 development framework at all levels to ensure that the framework includes a dedicated goal on universal access to WASH, and that the framework is adequately structured to reflect the need for cross-sectoral action by embedding WASH aspects in the proposed health goals and targets.
5. **Ensure adequate financial resourcing to WASH as a core health strategy:** the recognition of the importance of WASH as a determinant of MNH and a crucial part of MNH services should be reflected in terms of targeting and monitoring of financial resources. Resourcing should take into consideration not only the capital costs of infrastructure but also aspects of sustainability, accessibility, and affordability, at household and health care facility levels. These resources should include more and better-targeted investment in water and sanitation infrastructure in national budgets as well as a redoubling of efforts to meet access targets towards achieving universal access by 2030. Aid policy and financial channels should be adjusted to enable the use of aid resources to implement multi-sectoral and integrated MNH plans and programmes.

Many additional, specific actions can and should be taken by governments, health care providers, donors, the research community, and advocates from civil society and health care user groups. These are set out in Table 1.

**Table 1:** Policy recommendations.

Stakeholder	Recommendations
**All actors**	• Coordinate collection and publication of data on domestic and facility WASH access (health facility assessments, inspections, censuses, and surveys) for improved planning.• Use technology (GPS locations of facilities, crowd sourcing information on WASH in facilities) to complement data collection efforts.
**Governments of high-burden maternal and newborn mortality countries**	• **Invest:** Increase and better-target investment in WASH infrastructure; increase efforts to meet MDG access targets and progressively work towards achieving universal access by 2030.• **Create an enabling environment:**○ Set standards, legislation, indicators, and monitoring system for WASH provision and practice in health care facilities and engage in global discussions for such standards. Identify barriers and solutions to integration and cross sector collaboration and address these through improved policies, strategies, legislation, coordination mechanisms, and financial systems. Ensure financial allocation for capital and operational infrastructure expenditure.
**Donor community**	• Ensure that WASH targets and indicators are embedded in global maternal health frameworks, the UHC global monitoring framework, and within the post-2015 development framework.• Respond to the need for cross-sectoral action to achieve these targets by encouraging inclusion of an integrated framework for health, road and transportation, and sustainable water and sanitation services in the recipient country's development agenda and proposals. This should include inserting conditionality measures into funding proposals.• Create the necessary changes in aid policy and financial channels to enable adoption and scale up of the integrated approaches.• Use medium- and long-term improvements in health outcomes, rather than short term outputs, to assess programme success.
**Health care providers and managers**	• **Improve WASH provision and practices:**○ Provide equipment, investment, training, and collaboration for infection control protocols and supplies in public and private facilities. Apply simple, low-cost practices to maintain basic hygiene and sterile conditions, particularly in delivery rooms and operating theatres.○ Adopt guidelines on good WASH practices in the Infection Prevention and Control guidelines.○ Include WASH aspects within job descriptions and performance assessments of health staff; provide WASH training and accreditation; encourage staff to act as promoters towards mothers and families.• **Promote safe behaviours:**○ Distribute appropriate promotional materials for use by health workers, outreach personnel, and volunteers in communication with communities.○ Embed promotion of safe WASH practices in routine communication between health care providers and service users.○ Use community-based approaches such as mothers groups, WASH community mobilisation activities, and community health clubs to implement innovative hygiene and sanitation behaviour change approaches.
**Academia and research institutions**	• Build a stronger evidence base on the linkages between WASH and MNH through assessing effectiveness of interventions.• Develop further research regarding the cost-benefit and economic sustainability of an integrated framework for health, sustainable WASH, and other infrastructure services.• Develop research to address key knowledge gaps, namely:○ Understanding of WASH-related exposures in relation to MNH, to inform the definition of WASH-safe/unsafe environments, which will in turn improve instruments to assess WASH provision in health care settings and enhance monitoring;○ Assessing the impact of lack of WASH provision in health care facilities on demand-side aspects, such as user satisfaction, and levels of facility (versus home) births; and○ Assessing the impact of lack of WASH provision in health care facilities on the occupational safety, practices, and motivation levels of health care workers.
**Advocates, civil society and service users**	• Hold government and other agencies to account for delivering universal access to acceptable and dignified health services, and sustainable water and sanitation services.• Help define and deliver solutions.

## Conclusions

Many of the challenges highlighted in this paper can be seen as opportunities. The actions we propose are achievable and offer significant positive externalities beyond the health of mothers and newborns. The timing for action is favourable. The opportunity to develop an improved international development framework is one good reason. Another is the increased acceptance, demonstrated by the publication of this paper and the broad coalition of stakeholders that contributed to it, of the need for cross-sectoral action. The prospect of bolder and more ambitious goals on health and WASH replacing the existing MDG targets offers an opportunity that should not be missed to create a broad-based effort to address the slow progress on MNH and mortality, and help address the unequal burden of maternal and newborn mortality borne by high-burden countries, and the poorest and most at-risk populations globally.

Although further research is required to increase our understanding of the specific direct and indirect mechanisms that link WASH and MNH, to quantify the effects of particular interventions on specific maternal outcomes, and to assess the relative importance of different interventions in different settings, there is sufficient knowledge to justify action. The pursuit of further knowledge should be done in conjunction with, and not prior to, the actions proposed in this paper. While these links are complex and difficult to quantify, there should be no argument with the fact that women worldwide are entitled to clean, safe, and dignified environments during pregnancy, childbirth, and the postpartum period. It is also clear that any investment aimed at improving MNH through better WASH facilities at home or in health care facilities will yield positive externalities for the wider population, including children and other family members at home, and other patients and medical staff or care-givers in health care facilities. The neglect of this basic human right continues to frustrate global efforts to improve MNH.

We call on governments and other agencies to implement the measures described in this paper; and we call on health care staff and members of the public to demand universal access to acceptable and dignified health services, and sustainable, accessible, and affordable water and sanitation services. All of us must play our roles in securing a cleaner, safer, and healthier future for all mothers and newborns.
